# Genotype-Phenotype Correlations of β-Thalassemia Mutations in an Azerbaijani Population

**DOI:** 10.4274/tjh.2016.0427

**Published:** 2017-08-02

**Authors:** Chingiz Asadov, Eldar Abdulalimov, Tahira Mammadova, Surmaya Gafarova, Yegana Guliyeva, Gunay Aliyeva

**Affiliations:** 1 Department of Hereditary Pathology of the Erythrocyte System, Institute of Hematology and Transfusiology, Baku, Azerbaijan

**Keywords:** Thalassemia, Sickle/β-thalassemia, Codon, genotype, phenotype

## Abstract

β-Thalassemia is the most common inherited disorder in Azerbaijan. The aim of our study was to reveal genotype-to-phenotype correlations of the most common β-thalassemia mutations in an Azerbaijani population. Patients with codon 8 (-AA), IVS-I-6 (T>C), and IVS-II-1 (G>A) mutations, which are reportedly the most common β-globin gene mutations among the local population, were tested for hematologic parameters. Fifty-five previously tested patients with known genotypes were included in the study. Hematologic indices and hemoglobin fractions were tested in order to reveal the phenotypic manifestation of the mutations. The results obtained indicate that clinical presentation varies between different β-globin gene mutations: individuals with IVS-I-6 (T>C) mutations showed milder presentation than those with codon 8 (-AA) and IVS-II-1 (G>A), which is associated with the molecular basis of the mutations. These data can be of assistance to predict clinical presentation and select the best possible therapeutic approach via early genotype identification.

## Abstract (tr)

β-Talasemi Azerbaycan’da görülen en sık kalıtsal hastalıktır. Çalışmamızın amacı Azerbaycan popülasyonunda en sık görülen β-talasemi mutasyonlarının genotip-fenotip korelasyonlarının ortaya çıkarılmasıydı. Yerel popülasyonda en sık görülen β-globin gen mutasyonları olan kodon 8 (-AA), IVS-I-6 (T>C) ve IVS-II-1 (G>A) mutasyonlarını taşıyan hastalar hematolojik parametreler açısından değerlendirildi. Daha önceden test edilmiş ve genotipik özellikleri bilinen 55 hasta çalışmaya dahil edildi. Mutasyonların fenotipik manifestasyonlarının gösterilmesi için hematolojik indeksler ve hemoglobin fraksiyonlarına bakıldı. Sonuçlar farklı β-globin gen mutasyonları arasında klinik başvuru açısından farklılıklar olduğunu göstermekteydi: IVS-I-6 (T>C) mutasyonu olan bireylerde hastalık kodon 8 (-AA) ve IVS-II-1 (G>A) mutasyonu olanlara göre daha hafif seyretmekteydi. Erken genotipik tanımlama klinik başvuru özelliklerinin tahmin edilmesine ve hastalara en iyi terapötik yaklaşımların uygulanmasına yardımcı olabilir.

## INTRODUCTION

Hereditary hemoglobinopathies are the most common monogenic diseases. It is estimated that the frequency of carriers is 5.2% among the world population and there are over 330,000 births with hemoglobin disorders annually, of which 17% are thalassemias [1,2]. There are more than 200 mutations associated with β-thalassemias, and the spectrum and frequency of mutations varies significantly even in different regions of a single country [3,4]. Azerbaijan is one of the countries with the highest prevalence of thalassemias [5]. The frequency of carriers of β-thalassemia genes varies in different regions of the country from 0% to 17%, with a mean of 8.7% [6]. Structural hemoglobinopathies like sickle hemoglobin (HbS), HbD, HbC, and HbE are also detected in Azerbaijan and can be co-inherited with β-thalassemia [7,8].

β-Thalassemia is known for its extremely diverse clinical manifestations. It can be expressed mildly without a need for treatment, or in a severe form observed as profound anemia, hepatosplenomegaly, and significant deteriorations in bones that can be lethal during childhood if not treated appropriately [9,10]. Therefore, determination of factors causing such a diverse clinical presentation has clinical significance, and the major reason for such diversity is the variety of mutations [11,12,13,14,15,16]. A number of studies revealed genotype-to-phenotype correlations of β-thalassemia mutations in various populations [17,18,19], and we hereby report the first one conducted in an Azerbaijani population. Recent studies reported 22 mutations of β-thalassemia in Azerbaijan, and it was found that the most common mutations among the Azerbaijani population are codon 8 (-AA), IVS-I-6 (T>C), and IVS-II-1 (G>A) [7,20,21]. The aim of our study was to investigate phenotypic manifestations of these three mutations by studying their hematologic parameters and clinical presentation in heterozygous, homozygous, and compound forms. Our data will reveal the genotype-to-phenotype correlation of each mutation, providing a better prediction of the clinical manifestation of the disease by early genotype identification.

## MATERIALS AND METHODS

Fifty-five patients with known genotypes were included in the study. There were 23 heterozygous, 23 homozygous, 2 compound heterozygous, and 7 sickle β-thalassemia patients. Patients were chosen randomly; newly diagnosed transfusion-naïve patients as well as those already receiving therapy were included in the study. However, testing of hematologic parameters was done prior to transfusion. Written informed consent was obtained. Blood samples were collected into ethylenediaminetetraacetic acid-containing tubes. Evaluations of red blood cell (RBC) count, hemoglobin concentration, hematocrit, mean corpuscular volume (MCV), mean corpuscular hemoglobin (MCH), and mean corpuscular hemoglobin concentration (MCHC) were performed with a Sysmex XT2000i hematology analyzer (Sysmex, Kobe, Japan). All samples were tested for hemoglobin fractions by acetate-cellulose electrophoresis and high-performance liquid chromatography via the VARIANT IITM Hemoglobin Testing System (Bio-Rad Laboratories, Hercules, CA, USA). DNA amplification was performed on a C-1000 thermocycler (Bio-Rad). Detection of mutations by reverse dot-blot hybridization [11] was performed with commercial test kits (β-Globin StripAssay Kit, ViennaLab Cat. No. 4-130, ViennaLab Diagnostics, Vienna, Austria) according to the manufacturer’s instructions. One-way ANOVA followed by Tukey’s honestly significant difference test was used to analyze between-group differences.

## RESULTS

Hematologic data of heterozygous individuals are presented in Table 1. The classical β-thalassemia carrier pattern of increased RBC count and low hemoglobin and erythrocyte indices (MCV, MCH, and MCHC) was observed in almost all patients. Increased HbA2 concentrations and fetal hemoglobin within the normal range (<2%) were observed in most cases. There was a statistically significant difference (p<0.05) in RBC, MCV, and MCH parameters of IVS-I-6 patients compared to the groups with codon 8 and IVS-II-1. The former was associated with comparably lower RBC and higher MCV and MCH mean values. Based on this pattern of hematologic data, it can be concluded that IVS-I-6 presents a milder phenotype compared to codon 8 and IVS-II-1 mutations. The considerably lower hemoglobin level of one of the IVS-I-6 heterozygous patients was related to concomitant iron deficiency (No. 18; Hb=7.7 g/dL). No statistically significant difference was obtained between codon 8 and IVS-II-1 mutations (p>0.05).

The data of homozygous individuals were also observed to correlate with the type of the mutation (Table 2). Statistically significant between-group differences (p<0.05) were observed for MCV, MCH, and MCHC. Contrary to the heterozygous individuals, lower mean values of these parameters were observed in the group of homozygous IVS-I-6 patients, possibly due to their less frequent transfusions, compared to codon 8 and IVS-II-1 patients.

Table 3 presents the data of 7 patients with sickle β-thalassemia who have A>T substitution in the 6th codon (sickle cell mutation) together with codon 8 (-AA) mutation. RBC count, hematocrit, and erythrocyte indices (MCV, MCH, MCHC) were decreased and abnormal HbS was observed in all cases. The pattern of HbA2 and fetal hemoglobin was similar to that of the milder IVS-I-6 mutation, differing significantly from the other two (p<0.05). All patients were receiving recombinant erythropoietin and hydroxyurea therapy [22].

## DISCUSSION

From the chosen most common mutations of the Azerbaijani population, codon 8 (-AA) and IVS-II-1 are β^0^-mutations with complete absence of β-globin chain production, whereas IVS-I-6 is a β+-mutation, which is known to show decreased synthesis of β-globin protein. Codon 8 (-AA) is an RNA translation mutation located at the exon. Deletion of 2 adenine nucleotides at the 8^th^ codon (AAG-lysine) causes a frameshift mutation leading to an early termination at codon 21 (TGA). The new modified beta 8 (-AA) peptide does not function as a β-globin protein ending in β^0^-thalassemia. IVS-II-1 (G>A) and IVS-I-6 (T>C) are both RNA processing mutations located in the introns. The former is located in the splice junctions, causing impaired RNA processing, which ends in β+-thalassemia.

Knowledge of the molecular basis of the mutations elucidates the diversity of clinical presentations. β^0^-Mutations apparently have more severe clinical presentation, causing thalassemia major in the homozygous state, whereas β+-mutations are observed as milder cases of thalassemia intermedia. The same pattern was more clearly observed in heterozygous individuals with IVS-I-6 β+-mutation presenting comparably milder phenotypes (Table 1).

Our findings are mainly in concordance with the previous reports [3,23,24]. Hematologic parameters and clinical presentation correlate with the type of the mutation, considering the fact that the phenotypic manifestation of each mutation is directly related to its molecular basis. Nevertheless, concomitant diseases and non-genetic factors can also influence the clinical presentation of the disease, e.g., concomitant iron deficiency can considerably change hematologic parameters.

## CONCLUSION

Our study revealed genotype-to-phenotype correlations of the most prevalent β-thalassemia mutations of the Azerbaijani population. According to our data, hematologic parameters and consequently the clinical presentation are closely related to the type of the mutation, especially in homozygous patients. Our findings can provide a better prediction of clinical manifestation by early identification of the type of the β-thalassemia mutations.

## Figures and Tables

**Table 1:**
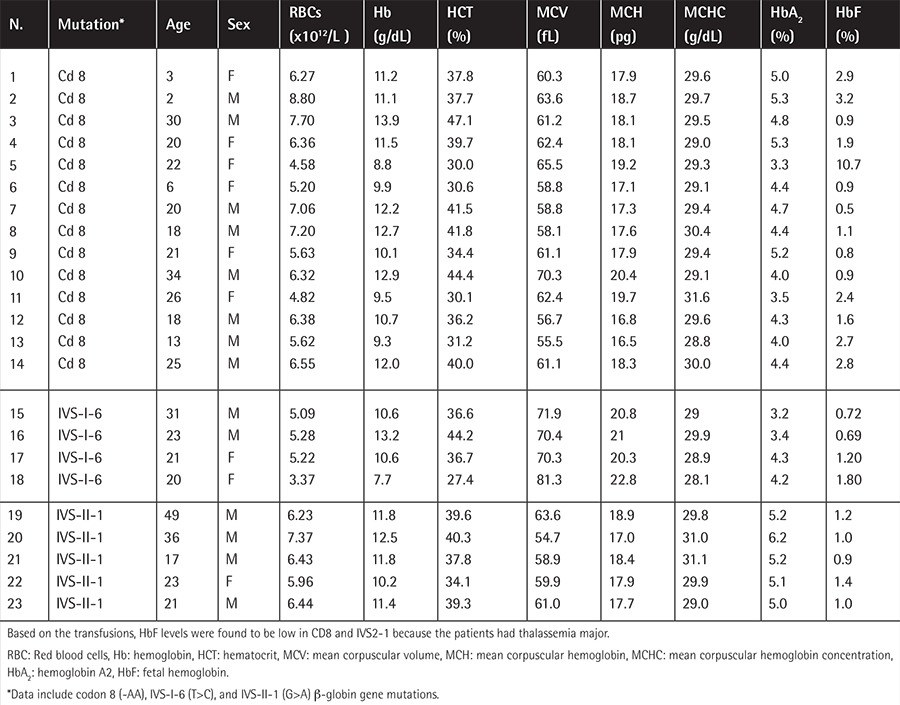
Hematologic data of heterozygous β-thalassemia patients.

**Table 2:**
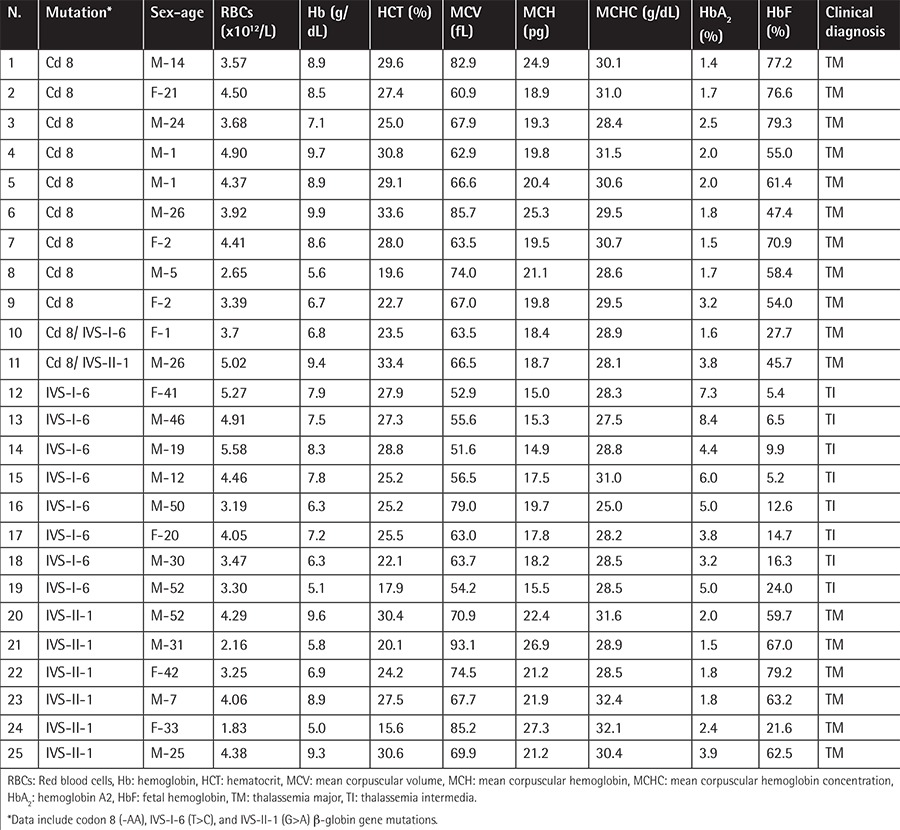
Hematologic data of homozygous and compound heterozygous β-thalassemia patients.

**Table 3:**
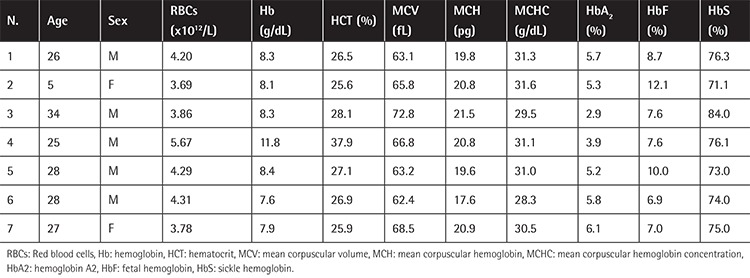
Hematologic data of sickle cell β-thalassemia patients with codon 8 (-AA) mutation.
